# Development and Evaluation of a Data Glove-Based System for Assisting Puzzle Solving

**DOI:** 10.3390/s26082341

**Published:** 2026-04-10

**Authors:** Shashank Srikanth Bharadwaj, Kazuma Sato, Lei Jing

**Affiliations:** The Graduate School of Computer Science and Engineering, The University of Aizu, Fukushima 965-8580, Japan

**Keywords:** data glove, tactile sensing, e-textile, Tower of Hanoi, human–computer interaction, step-by-step guidance, task verification, convolutional neural network

## Abstract

Many hands-on tasks remain difficult to fully automate because they require human dexterity and flexible object handling. Data gloves offer a promising interface for sensing hand–object interactions, but most prior systems focus on gesture recognition or object classification rather than closed-loop, step-by-step task guidance. In this work, we develop and evaluate a tactile-sensing operation support system using an e-textile data glove with 88 pressure sensors, a tactile pressure sheet for placement verification, and a GUI that provides step-by-step instructions. As a core component, a CNN classifies the grasped state as bare hand or one of four discs with 93.3% accuracy using 16,175 training samples collected from five participants. In a user study on the Tower of Hanoi task as a controlled proxy for multi-step manipulation, the system reduced mean solving time by 51.5% (from 242.6 s to 117.8 s), reduced the number of disc movements (35.4 to 15, about 20 fewer moves on average), and lowered perceived workload (NASA-TLX) by 53.1% (from 68.5 to 32.1), while achieving a SUS score of 75. These results demonstrate the feasibility of tactile-based step verification and guidance in a controlled multi-step task; broader generalization requires evaluation with larger and more diverse participant groups and tasks.

## 1. Introduction

Many hands-on tasks remain difficult to fully automate because they require human adaptability, fine manipulation, and contextual judgment in nonstandard situations. While AI-enabled systems have achieved strong performance in domains such as digital transformation, smart manufacturing, autonomous systems, anomaly detection, and fault diagnosis [1,2,3,4,5], fully automated deployment can be constrained by environment-specific variability and the cost of specialized equipment. Therefore, approaches that support human operators—rather than replacing them—remain important for multi-step manipulation tasks.

Wearable sensing technologies have emerged as a complementary approach for supporting human operators without fully replacing them. Among these technologies, tactile sensing systems and sensorized surfaces have demonstrated the feasibility of capturing contact information and interaction patterns [6,7]. Data gloves have gained considerable attention due to their ability to capture hand posture, finger movement, grasp patterns, and tactile interaction. Typical data glove systems integrate tactile sensors, flex sensors, or inertial measurement units (IMUs) and have been applied to gesture recognition, object identification, virtual and augmented reality control, and motion tracking [8,9,10,11,12,13]. Although these systems demonstrate strong sensing performance, most function primarily as measurement devices, and relatively little work has examined how data gloves can provide real-time procedural task guidance and verification.

Operation support systems using smart glasses and extended reality (XR) technologies offer visual overlays, procedural instructions, and object detection through camera-based perception [14,15,16]. While effective in many settings, vision-based systems can suffer from occlusion, blind spots, and sensitivity to lighting conditions, and they require reliable camera placement. Tactile and wearable sensor systems avoid these limitations by directly detecting hand–object interactions, making them suitable for tasks where visual sensing is unreliable.

To explore how tactile sensing can contribute to interactive task assistance, this work uses the Tower of Hanoi puzzle as a controlled proxy task. The Tower of Hanoi is widely used in human factors and cognitive science because it has a deterministic optimal solution and requires sequential reasoning [17,18]. Importantly, our goal is not to outperform conventional keyboard-based input for solving the puzzle, but to evaluate a closed-loop guidance and verification pipeline for hands-busy, multi-step manipulation under controlled conditions.

In this study, we develop an operation support system built around an e-textile tactile data glove. The glove incorporates 88 pressure sensors distributed across the fingers and palm, and the setup includes a tactile pressure sheet placed underneath the puzzle stand. A convolutional neural network (CNN) classifies the grasped object as bare hand or one of four disc sizes based on tactile patterns. A graphical user interface (GUI) provides step-by-step instructions, visual animations, and real-time feedback. The system verifies each move by jointly analyzing glove and pressure-sheet data, enabling users to follow the optimal sequence.

To evaluate the system, we conducted a controlled user study comparing a baseline group solving the puzzle without assistance and a support group using the proposed system. Objective task performance was measured using solving time and number of disc movements. Subjective workload was assessed with the NASA Task Load Index (NASA-TLX), and usability was evaluated using the System Usability Scale (SUS). The results show that the system reduces solving time and unnecessary movements, lowers perceived workload, and achieves a SUS score indicative of good usability; however, broader generalization and real-world deployment would require additional evaluation with more diverse participants and tasks.

The remainder of this paper is organized as follows: Section 2 reviews related research. Section 3 describes the tactile data glove, pressure sheet, and overall system structure. Section 4 explains the data collection procedures, preprocessing, and experimental protocol. Section 5 presents the CNN model. Section 6 reports the experimental results. Section 7 discusses implications and limitations. Section 8 concludes the paper.

## 2. Related Work

Wearable sensing research has expanded significantly in recent years, particularly in the development of data gloves and operation support frameworks that combine human–computer interaction with recognition models. This section reviews representative studies on tactile and inertial data gloves, followed by work on operation support systems using smart glasses and XR. We then summarize key gaps and explain how the present study addresses them.

### 2.1. Data Gloves and Tactile Sensing

Data gloves have been widely explored as wearable interfaces for capturing hand posture, finger joint motion, and tactile interaction. Early systems relied primarily on flex sensors or IMUs to reconstruct hand poses or gestures, enabling real-time gesture recognition and hand trajectory visualization [8,9,10]. More recent approaches integrate dense tactile sensor arrays that detect pressure distributions across the hand surface, allowing for fine-grained recognition of grasp patterns and object characteristics.

Sundaram et al. introduced a high-resolution tactile glove containing over 500 sensors, capable of recognizing everyday objects and estimating object properties using a CNN [8]. Pohtongkam and Srinonchat compared CNN-based object recognition with a Bag-of-Words (BoW) approach and showed that although BoW offered faster computation, CNN models achieved substantially higher accuracy [11].

Other studies combined IMUs with tactile sensors to capture both finger motion and contact information. Liu et al. constructed a glove with IMUs and tactile sensors for visualization of hand posture and pressure distribution during manipulation [9]. Makaussov et al. presented a low-cost IMU glove for real-time gesture detection [12]. Tashakori et al. integrated textile-based sensing with IMUs for complex hand motion and object interaction capture [13]. Additional fabric-based bending sensors and glove applications have also been reported [19,20]. A broader review of glove-based sensing in health/rehabilitation contexts was provided by Henderson et al. [21].

Across these studies, the primary emphasis is sensing accuracy and recognition performance. However, most glove-based systems function as passive input devices, and relatively few works explore how tactile glove data can provide active, step-by-step task support with verification of user actions.

### 2.2. Operation Support Using Smart Glasses and XR

Operation support systems often rely on visual displays to guide users through complex tasks. Smart glasses have been used to provide context-aware instructions and object detection through camera-based perception [14]. In industrial settings, Silva et al. demonstrated smart-glasses-based inspection using real-time defect detection [15]. XR environments have also been used for operator training and skill acquisition in simulated settings [16].

However, vision-based systems can suffer from occlusion, blind spots, camera mounting constraints, and sensitivity to lighting variations. These issues restrict reliability in environments where hand–object interactions are not consistently visible. Wearable tactile systems avoid these failure modes by directly capturing contact patterns at the hand.

### 2.3. Comparison of Representative Approaches

Table 1 summarizes representative glove-based sensing and operation support approaches and highlights that many prior works emphasize recognition (gesture/object) or camera-based guidance, while fewer integrate tactile sensing into a closed-loop verification and guidance pipeline.

Recent tactile-glove and tactile-textile studies primarily focus on recognition or estimation (e.g., object identification and interaction modeling) [8,11,22], while vision/XR approaches emphasize visual overlays and camera-based detection for guidance [14,15,16]. In contrast, our contribution is a closed-loop pipeline that combines (i) tactile grasp-state recognition from the glove with (ii) an independent placement-verification channel via a pressure sheet, and uses both signals to verify each step and provide procedural guidance in a deterministic multi-step task (Tower of Hanoi as a controlled proxy).

### 2.4. Summary of Gaps and Design Implications

Based on the above literature, we identify the following gaps relevant to tactile-based task assistance:

- Recognition vs. guidance: Many data-glove studies focus on gesture/object recognition, but do not couple recognition with action verification and step-by-step procedural support.
- Reliance on vision: Smart glasses and XR systems provide rich visual guidance but can be affected by occlusion and lighting, particularly when hand–object contact is difficult to observe.
- Limited closed-loop verification: Few systems explicitly verify whether a user executed the correct step using multimodal contact information (e.g., grasp state plus placement confirmation).

These gaps motivate a system design that (i) uses tactile sensing to infer grasped objects, (ii) uses an additional contact channel (pressure sheet) to verify placement, and (iii) integrates both into a closed-loop GUI that guides users through an optimal multi-step sequence. We evaluate this concept using the Tower of Hanoi as a controlled proxy task for multi-step manipulation.

### 2.5. Positioning of This Work

The present study bridges tactile data glove sensing and interactive operation support. Unlike most prior data glove work, which focuses on gesture or object recognition [8,9,10,11,12,13], our goal is not merely to classify hand states but to embed tactile sensing into an interactive support pipeline that verifies user actions and provides procedural guidance. Compared with smart glasses and XR-based systems [14,15,16], the proposed method does not rely on external cameras, making it robust to occlusion and lighting. To the best of our knowledge, prior work has less frequently combined tactile grasp-state recognition with an independent placement-verification channel into a single closed-loop system for step verification and procedural guidance in multi-step manipulation. This motivates the development and evaluation of the system presented in this paper.

## 3. System Description

This section describes the key components of the proposed operation support system: (1) the tactile data glove, (2) the tactile pressure sheet, (3) the Tower of Hanoi task environment, and (4) the overall system architecture and GUI.

### 3.1. Tactile Sensor and Data Glove

**Figure 1:**
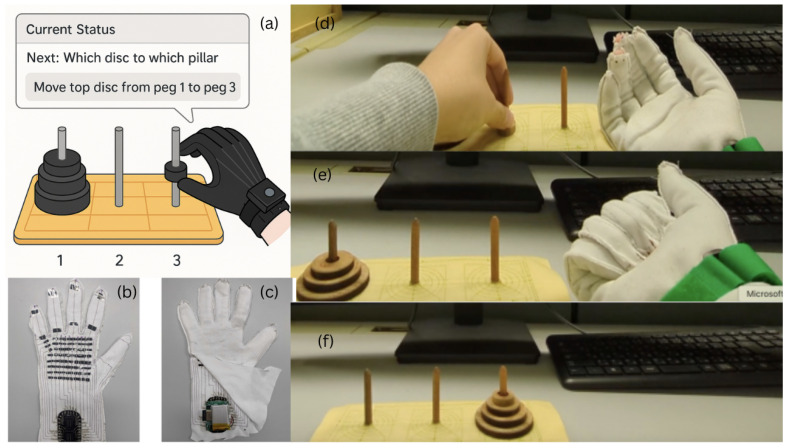
System overview and user interaction sequence. (**a**) GUI instruction example for the Tower of Hanoi. (**b**,**c**) Tactile glove prototype (sensor side and controller side). (**d**–**f**) Representative interaction sequence during disc transfer on the Tower of Hanoi setup.

The tactile data glove is constructed using an e-textile sensor array embedded into a flexible fabric substrate. Each tactile unit consists of two conductive fabric layers separated by a compressible resistive material. When pressure is applied, the resistance decreases, enabling the detection of contact forces at various locations on the hand. A total of 88 tactile sensors are distributed across the palm and fingers to capture grasp patterns and object-contact information. Figure 1 shows the overall system overview and representative user interaction sequence.

**Figure 2:**
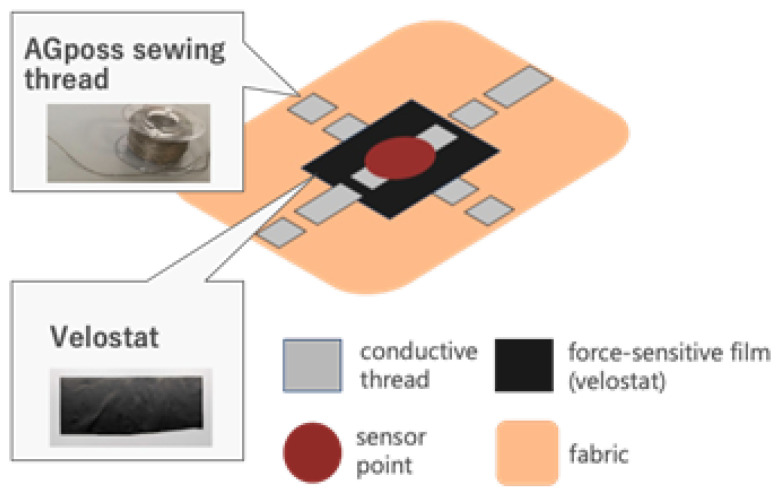
Basic structure of the e-textile tactile sensor used in the data glove. Pressure reduces electrical resistance between the conductive layers.

The basic structure of the e-textile tactile sensor is shown in Figure 2.

**Figure 3:**
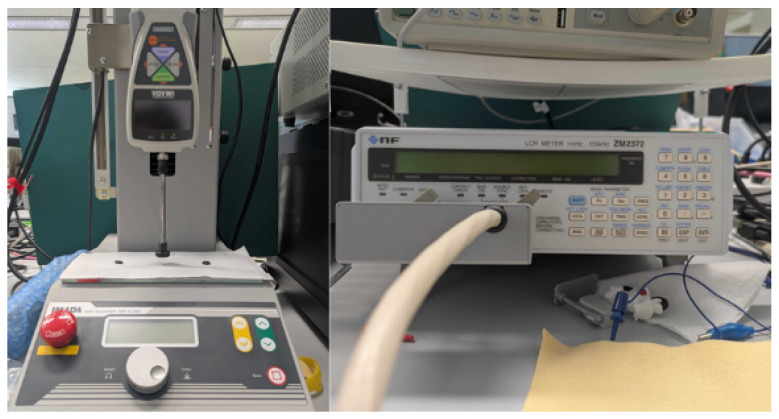
Experimental setup for characterizing the tactile sensor using a force gauge and an LCR meter.

**Figure 4:**
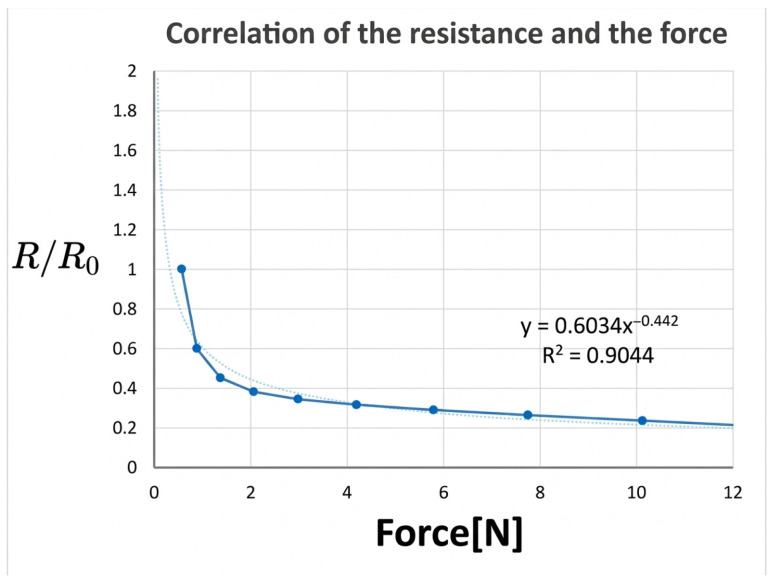
Measured force–resistance characteristics of the e-textile tactile sensor under quasi-static loading. The curve shows a representative sensing element; sensor-to-sensor variability due to fabrication tolerances is discussed as a limitation in Section 7.

To characterize the tactile sensing behavior, a force gauge and an LCR meter were used to measure the relationship between applied force and resistance. The experimental setup is shown in Figure 3. These measurements confirmed that the sensors exhibit a monotonic decrease in resistance with increasing force, enabling effective pressure estimation. Representative force–resistance characteristics are shown in Figure 4.

While the response is monotonic, individual sensing elements can exhibit offset and slope variation due to fabrication tolerances.

**Figure 5:**
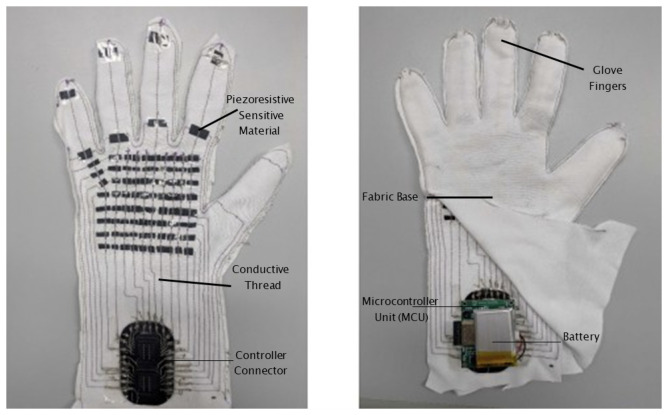
Completed tactile data glove. A total of 88 tactile sensors are embedded across the palm and fingers (80 on the palm region and 8 on the finger region), as summarized in Table 2.

The data glove was designed to match the anatomical contours of the user’s hand. Sensor placement was optimized to ensure that grasp interactions with puzzle discs could be captured reliably. The completed glove is shown in Figure 5.

**Table 2:** Region-wise placement of the 88 tactile sensors on the glove.

Region	Sensor Count	Rationale (Disc Interaction)
Fingers (combined)	8	Detect fingertip/finger contact cues during grasp initiation
Palm (combined)	80	Capture pressure distribution during disc support and transport
Total	88	

Sensors are concentrated on the palm to capture stable pressure patterns during disc support, with a smaller set on the fingers to capture contact cues during grasp initiation.

**Figure 6:**
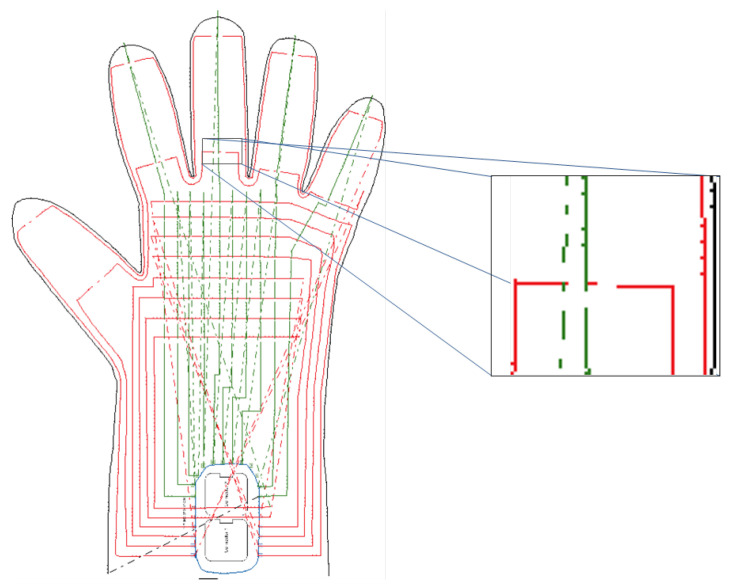
Glove fabrication template used to embed the tactile array: sewing pattern for the fabric substrate and the intended placement regions of tactile elements prior to stitching.

The glove’s sewing pattern and tactile array layout were determined based on the hand size and the dimensions of the Tower of Hanoi discs. The fabrication template is shown in Figure 6.

This template was used during fabrication to ensure consistent alignment between the glove structure and the tactile sensor placement.

**Figure 7:**
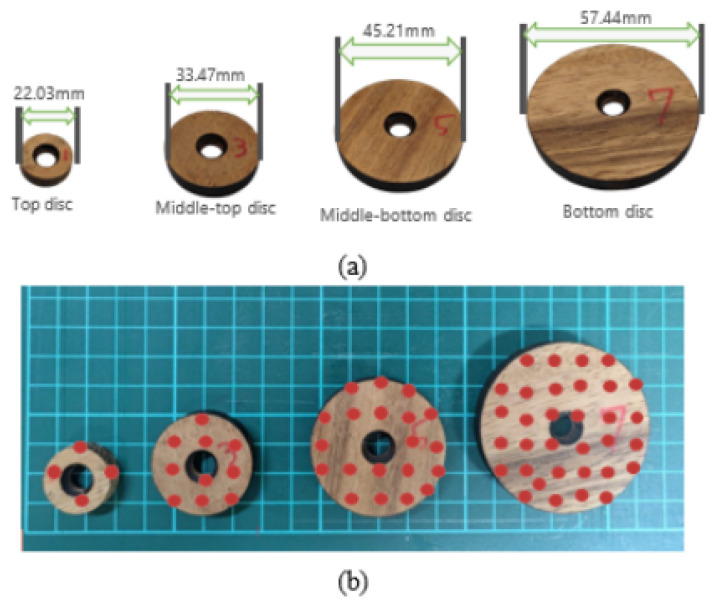
Disc sizes and corresponding sensor regions on the glove that capture pressure signatures during grasping. (**a**) Disc sizes used in the Tower of Hanoi puzzle. (**b**) Corresponding sensor regions on the glove associated with disc grasping. The red circles indicate representative contact regions.

To capture grasp-specific pressure signatures, the sizes of the discs used in the Tower of Hanoi puzzle were analyzed, and representative contact regions were mapped to sensor positions on the glove, as shown in Figure 7.

**Figure 8:**
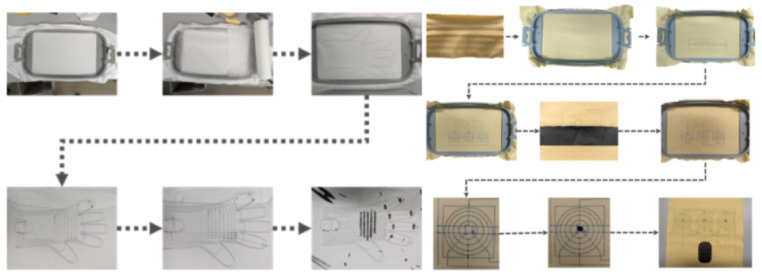
Fabrication process for the tactile glove and pressure sheet, including embroidery, assembly, and electronics integration.

The fabrication process for the tactile glove and pressure sheet includes embroidery of conductive threads, layering of resistive fabric, thermal bonding, and electronics integration, as shown in Figure 8.

**Figure 9:**
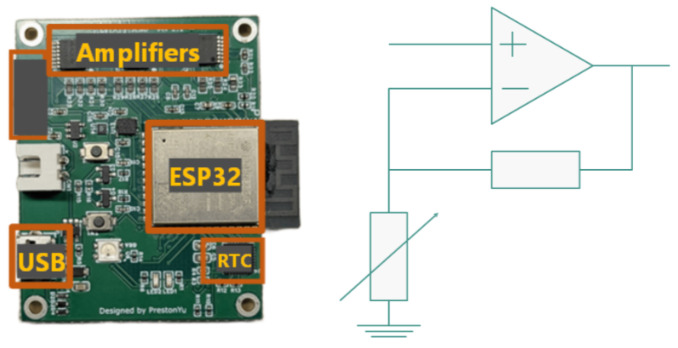
Controller board and amplifier circuit used for reading tactile sensor values from the glove.

The glove interfaces with a compact controller board containing an analog front end and a non-inverting amplifier. Sensor signals are digitized and transmitted to the processing unit. The controller board and amplifier circuit are shown in Figure 9.

### 3.2. Tower of Hanoi Task

**Figure 10:**
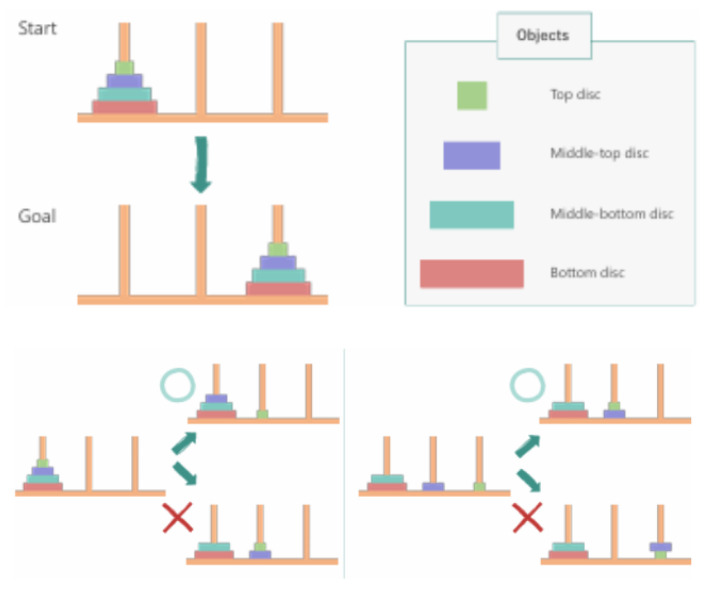
Tower of Hanoi task setup and puzzle rules used in this study.

The Tower of Hanoi puzzle consists of three pegs and a set of discs with decreasing diameters. The objective is to move the stack of discs from the initial peg to a target peg, following two rules: (1) only one disc may be moved at a time, and (2) no disc may be placed on top of a smaller disc. For *n* discs, the optimal solution requires $2^{n}-1$ moves. The task setup used in this study is shown in Figure 10.

### 3.3. System Architecture and Graphical User Interface

**Figure 11:**
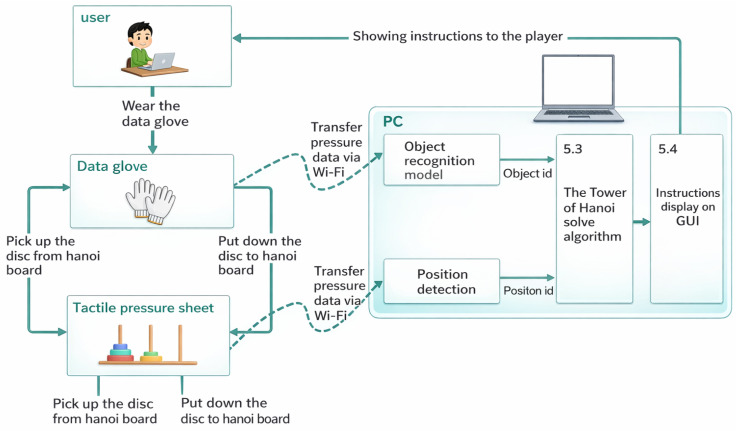
Overall architecture of the operation support system, including tactile sensing, object recognition, and instruction delivery.

The operation support system integrates the tactile data glove, tactile pressure sheet, CNN-based object classifier, and a real-time task guidance interface. Sensor data are transmitted to the processing unit, which detects whether the user has grasped a disc, identifies the disc size, and verifies whether the disc placement follows the optimal sequence. The GUI then displays animations and instructions that guide the user through each step of the puzzle. The overall architecture is shown in Figure 11.

**Figure 12:**
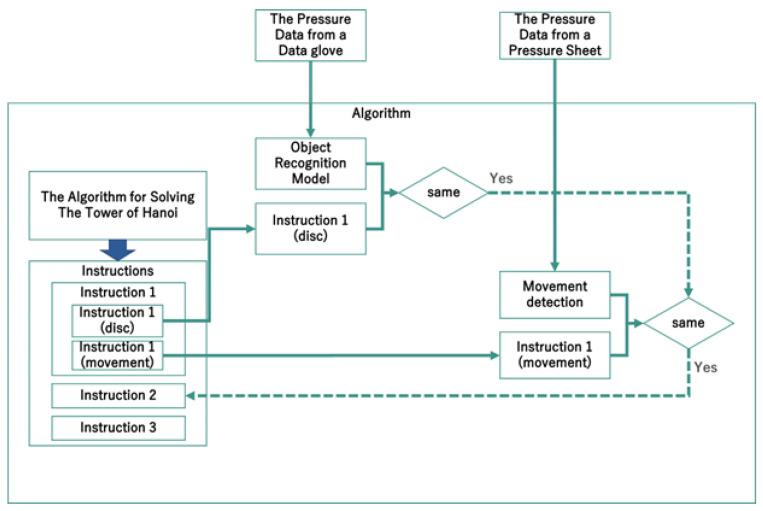
Algorithm flow summarizing disc detection, classification, validation of user action, and instruction generation.

**Figure 13:**
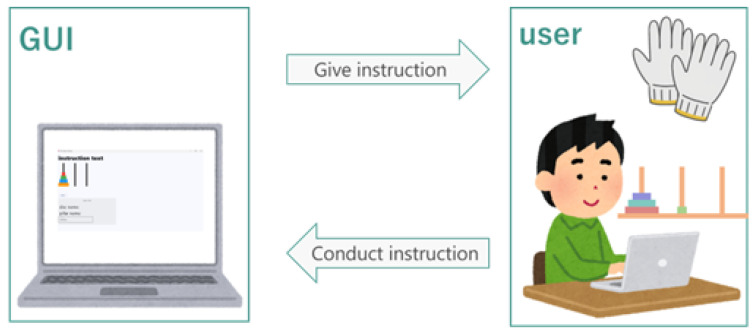
Relationship between the user, the tactile glove, the tactile sheet, and the GUI guidance system.

**Figure 14:**
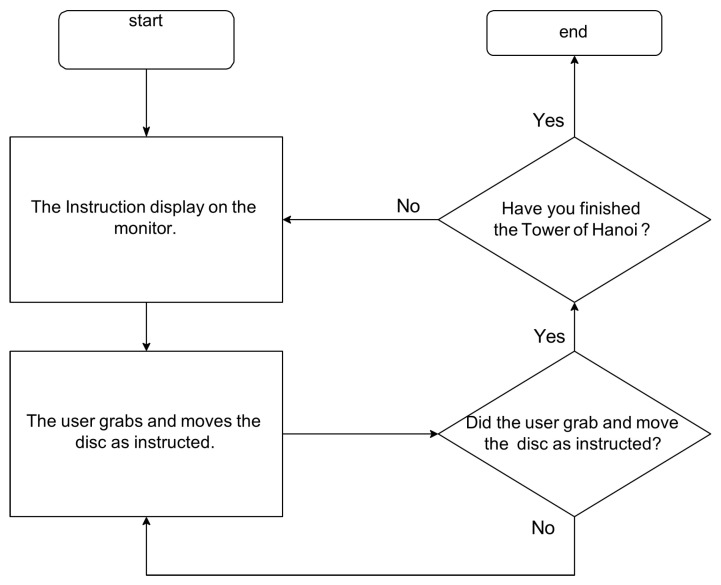
System flowchart illustrating sensing, interpretation, and instruction processes.

**Figure 15:**
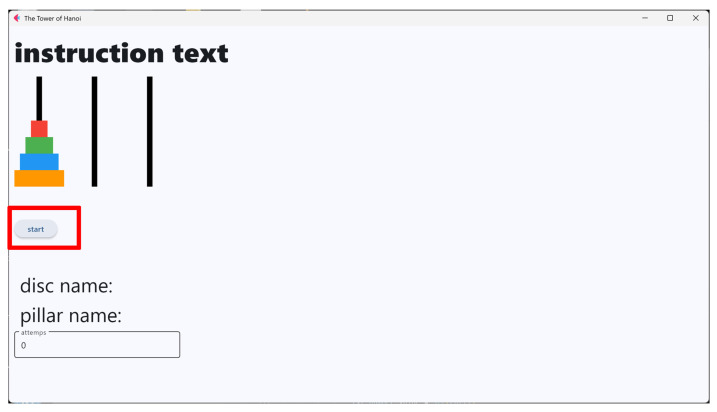
Overview of the GUI displaying instructions, disc states, and animations.

**Figure 16:**
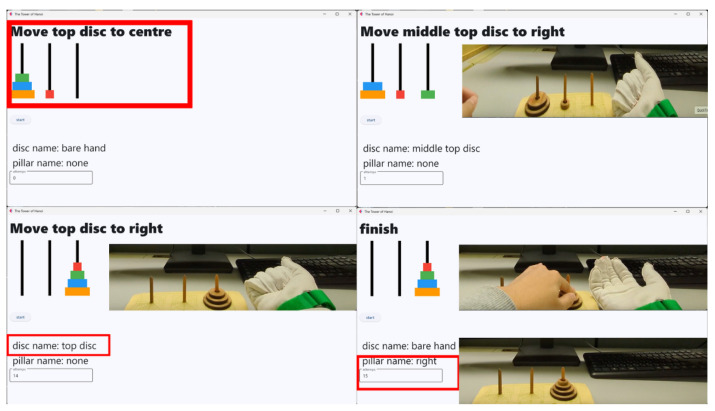
Representative GUI sequence showing step-by-step task guidance during the Tower of Hanoi puzzle.

Figure 12, Figure 13, Figure 14, Figure 15 and Figure 16 provide complementary views of the pipeline: logic flow, system-level processing, and GUI-level user interaction.

The GUI provides step-by-step instructions, visual cues, disc animations, and notifications when incorrect moves are attempted. An overview of the GUI is shown in Figure 15, and a representative sequence is shown in Figure 16.

## 4. Methods

This section describes the procedures used to construct the dataset, preprocess the pressure data, and annotate the recordings for training and evaluating the object-recognition model.

### 4.1. Dataset

#### 4.1.1. Data Collection

**Figure 17:**
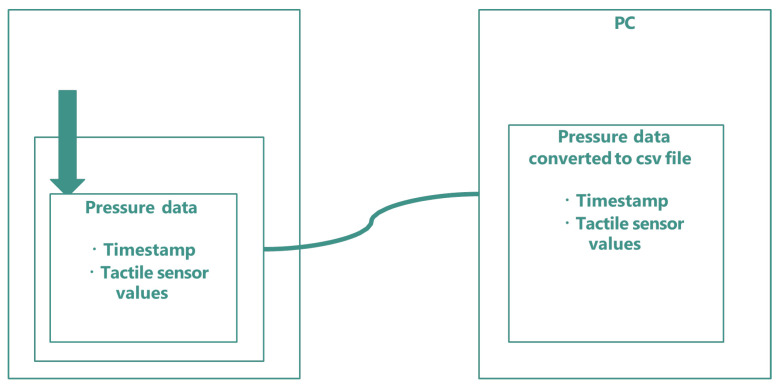
Overview of the data collection process. The data glove controller acquires tactile values from the glove, transmits the data to a PC via Wi-Fi using UDP, and stores timestamps and pressure values as CSV files.

Pressure data were collected using the tactile data glove described in Section 3.1 and the data glove controller. The controller sampled the glove at 10 Hz and transmitted the data to a PC via Wi-Fi using UDP communication. Each frame consisted of a timestamp and the values of all tactile elements, stored as a row in a CSV file. Figure 17 shows an overview of the data collection process.

To build an object-recognition dataset, participants grasped the Tower of Hanoi discs and formed a bare-hand pose without any disc. Four disc sizes were used (top, middle-top, middle-bottom, and bottom discs), and an additional “bare hand” condition represented the absence of a disc. For each disc, participants repeatedly grasped and released the disc while keeping it above the pressure sheet. For the bare-hand condition, participants repeatedly opened and closed the hand without holding a disc.

For each of the four discs, grasping sequences were recorded six times for 10 s per sequence. Bare-hand data were collected for 60 s, so that the number of samples for the bare-hand class was comparable to that of the disc classes. Pressure data and synchronized video were captured simultaneously to support later annotation.

**Figure 18:**
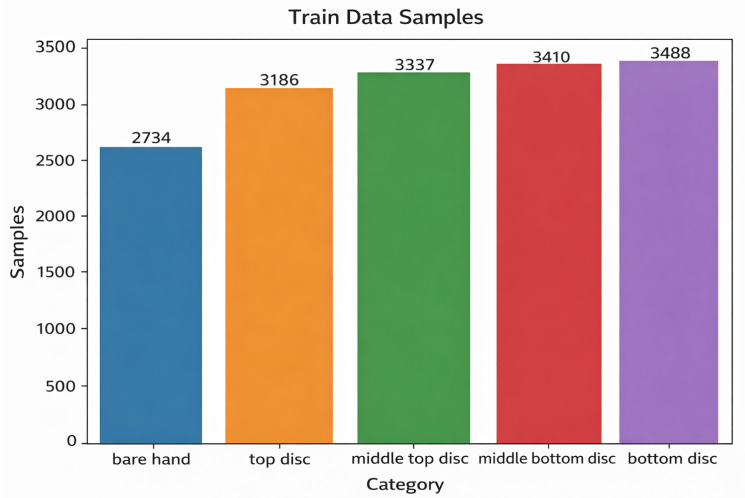
Class counts of the training dataset (*n* = 16,175), collected from five participants for bare hand and four disc sizes.

The final training set contained *n* = 16,175 samples: 2734 bare-hand instances, 3186 top-disc instances, 3337 middle-top-disc instances, 3430 middle-bottom-disc instances, and 3488 bottom-disc instances. A total of five individuals from the laboratory participated in the data collection under the same protocol. The class counts of the training dataset are shown in Figure 18.

**Figure 19:**
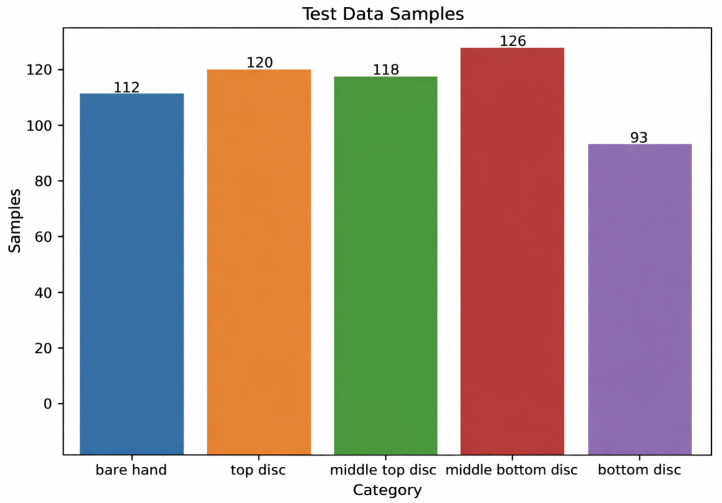
Class counts of the test dataset (*n* = 569) collected on a different day from a single participant.

A separate test set was collected on a different day from a single participant who had also contributed to the training data, primarily to evaluate day-to-day repeatability for a known user rather than cross-user generalization. For each class, pressure data were recorded for 10 s using the same protocol as in the training phase. The resulting test set contained *n* = 569 samples: 112 bare-hand instances, 120 top-disc instances, 118 middle-top-disc instances, 126 middle-bottom-disc instances, and 93 bottom-disc instances. The class counts of the test dataset are shown in Figure 19. Cross-user generalization is discussed as a limitation in Section 7.

#### 4.1.2. Data Structure

Each recorded sequence is stored as a CSV file with two fields per frame: a timestamp and a vector of tactile sensor values. After collection, each grasping episode is segmented into individual frames and associated with its class label. For CNN training, each frame is treated as an independent sample representing the instantaneous pressure distribution across the glove.

### 4.2. Data Processing

The raw pressure measurements from the e-textile glove include both disc-related forces and background grasping forces. To emphasize the contact patterns specific to each disc, a denoising procedure was applied based on pressure recordings of grasping motions without discs.

**Figure 20:**
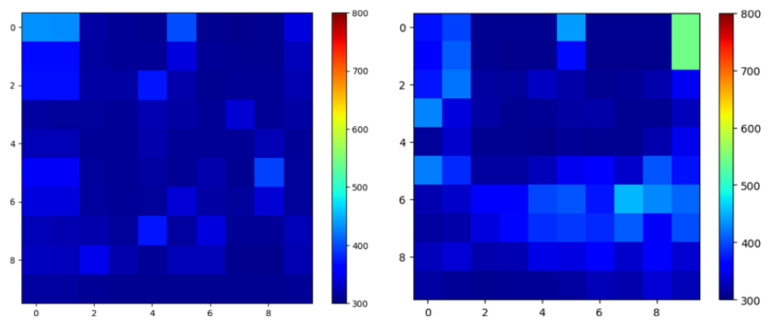
Example pressure maps from the tactile data glove while opening and closing the hand without a disc. These data are used to compute the average grasping-pressure distribution for denoising.

First, three types of grasping motions without a disc were recorded from five participants: opening the hand, closing the hand, and maintaining a light grasp. These sequences were used to compute an average grasping-pressure map for each sensor element. In total, 2283 frames of “no-disc” grasping data were collected. Example pressure maps from these motions are shown in Figure 20.

**Figure 21:**
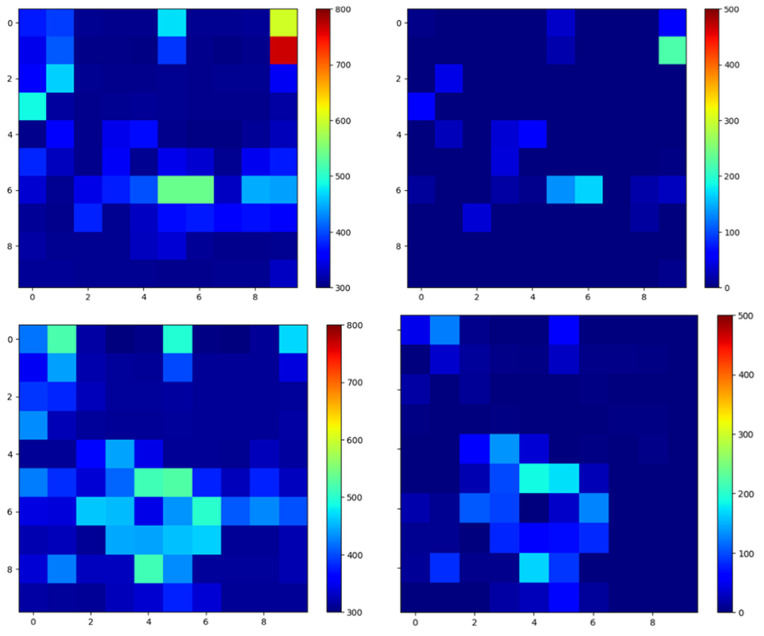
Pressure distributions when grasping each disc. (**Left column**): raw pressure maps including grasping and disc contact. (**Right column**): denoised maps obtained by subtracting the averaged no-disc grasping pressure, highlighting disc contact regions.

For each disc class, two pressure maps were computed: (1) the raw pressure map while grasping the disc and (2) the denoised pressure map obtained by subtracting the average no-disc grasping map. This subtraction attenuates the grasping component and highlights the contact region caused by the disc. The resulting pressure distributions are shown in Figure 21.

After denoising, each pressure vector is reshaped into a two-dimensional grid matching the physical layout of the tactile array. These 2D maps are used as input images for the CNN classifier described in Section 5.

### 4.3. Labeling and Quality Control

Rather than labeling every frame manually, we annotated continuous time intervals corresponding to stable grasps using synchronized video and pressure-map playback. For each labeled interval, the corresponding frames were assigned a class label (bare hand, top disc, middle-top disc, middle-bottom disc, or bottom disc). Ambiguous segments (e.g., incomplete grasps or unintended contacts) were excluded during verification. Detailed annotation-interface screenshots and implementation details were omitted from the main text to maintain focus on the model-development and evaluation pipeline.

### 4.4. Sample Preparation for Model Training

After annotation and preprocessing, each labeled frame is converted into a 2D pressure map with fixed spatial resolution. The following steps are applied:

1. Frame selection: For each annotated grasping interval, frames corresponding to the stable grasp phase are selected while transient frames at the start and end of the motion are discarded.
2. Normalization: Pressure values are normalized on a per-sensor basis to the range [0, 1] to reduce the impact of inter-sensor gain differences.
3. Reshaping: The normalized vector is reshaped into a 2D grid consistent with the physical layout of the 88 tactile elements.
4. Dataset split: Labeled samples are split into training and validation sets. The test set described in Section 4.1.1 is reserved exclusively for final evaluation.

The resulting dataset of 2D pressure maps serves as input to the CNN-based recognition model, which is described in detail in Section 5.

## 5. CNN-Based Tactile Object Recognition

This section describes the convolutional neural network (CNN) model used to classify the grasped object from the tactile pressure maps. The goal is to categorize each frame into one of five classes: bare hand, top disc, middle-top disc, middle-bottom disc, or bottom disc.

### 5.1. Input Representation

As described in Section 4.2, each tactile frame is converted into a two-dimensional pressure map that reflects the spatial layout of the 88 tactile elements. The resulting map is treated as a single-channel grayscale image. Let $X\in R^{H\times W}$ denote the normalized pressure map, where *H* and *W* are the height and width of the sensor grid.

The CNN operates on these 2D maps and learns spatial filters that respond to characteristic contact patterns for each disc size or the bare-hand condition. No hand-crafted features are used; all feature extraction is performed by the convolutional layers.

### 5.2. Network Architecture

**Figure 22:**
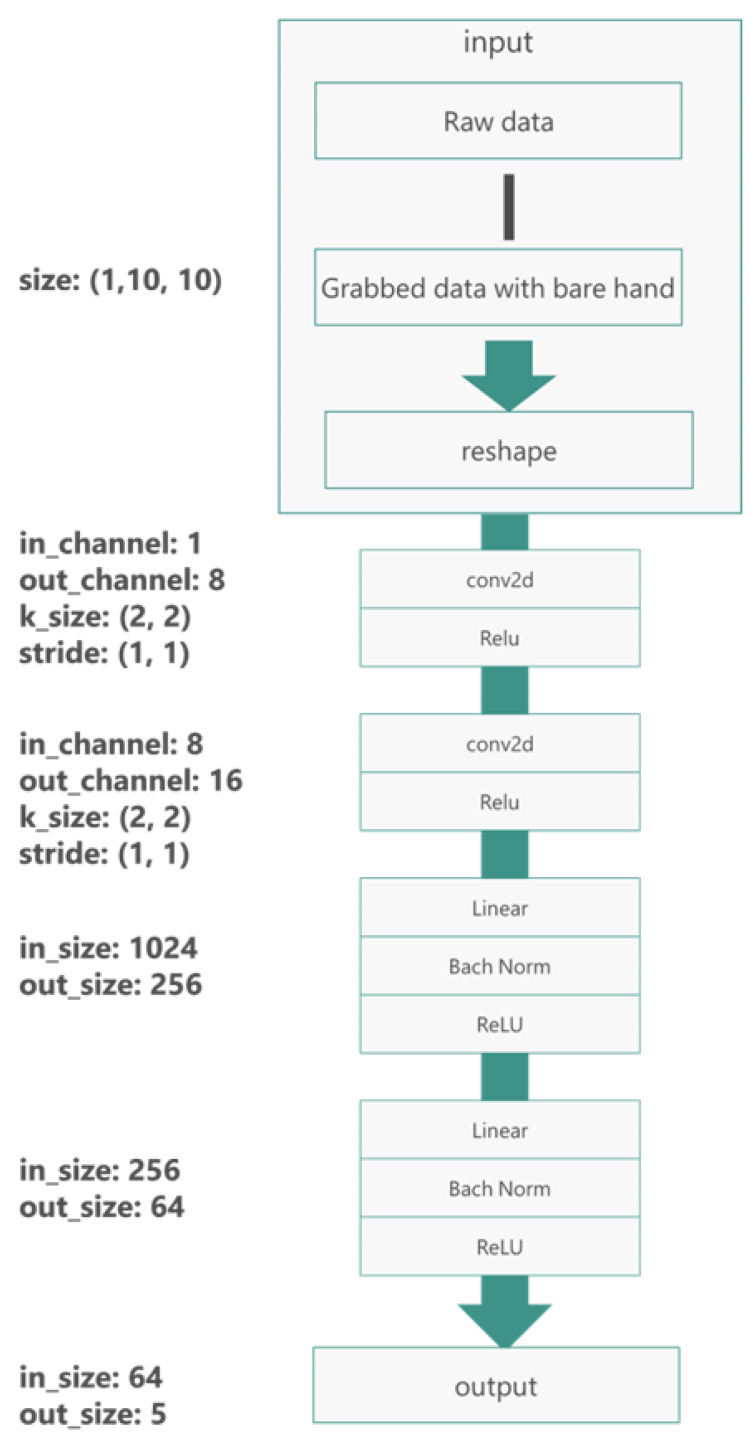
Architecture of the CNN used for object classification from tactile pressure maps. The network consists of three convolutional blocks followed by two fully connected layers and a softmax output layer.

The CNN architecture is summarized in Figure 22. It consists of three convolutional blocks followed by two fully connected layers and a softmax output layer. Each convolutional block includes a convolution layer, batch normalization, a rectified linear unit (ReLU) activation, and a max-pooling layer.

Concretely, the layers are defined as follows:

- **Conv Block 1:** 32 filters of size 3 × 3, stride 1, padding 1; batch normalization; ReLU; 2 × 2 max pooling.
- **Conv Block 2:** 64 filters of size 3 × 3, stride 1, padding 1; batch normalization; ReLU; 2 × 2 max pooling.
- **Conv Block 3:** 128 filters of size 3 × 3, stride 1, padding 1; batch normalization; ReLU; 2 × 2 max pooling.
- **Fully Connected 1:** 256 units with ReLU and dropout.
- **Fully Connected 2:** 5 output units corresponding to the five classes, followed by a softmax layer.

The number of units in the first fully connected layer is chosen so that the flattened feature map from the last convolutional block is fully connected without excessive parameters. Dropout with a probability of 0.5 is applied after the first fully connected layer to reduce overfitting.

### 5.3. Training Procedure

The model parameters are optimized using the cross-entropy loss function. Let y∈{1,…,5} denote the ground truth label for a sample, and let ${\hat{p}}_{k}$ be the predicted probability for class *k*. The loss for a single sample is

(1) $$L=-\sum_{k=1}^{5}I\left[y=k\right]\log{\hat{p}}_{k},$$

where I[·] is the indicator function.

**Figure 23:**
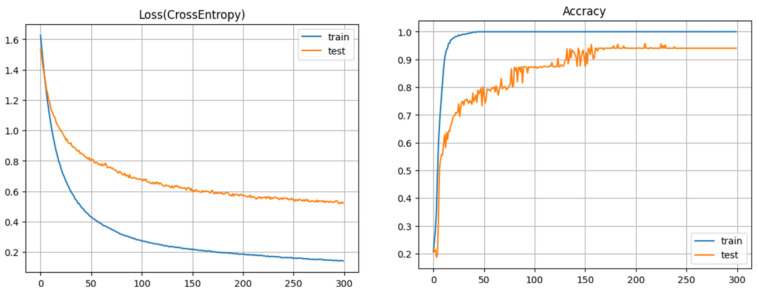
Training and validation curves of the CNN model. (**Right**): classification accuracy. (**Left**): cross-entropy loss. The model used for evaluation is selected at the epoch with minimum validation loss.

The network is trained using the Adam optimizer with an initial learning rate of $1\times 10^{-3}$, mini-batch size of 64, and weight decay for regularization. The learning rate is reduced when the validation loss stops improving. Training is performed for up to 100 epochs, and the model with the lowest validation loss is selected as the final model. Figure 23 shows the training and validation accuracy and loss over epochs.

### 5.4. Evaluation Metrics

The trained CNN is evaluated on the independent test set described in Section 4.1.1. Classification performance is quantified using overall accuracy and a confusion matrix, which summarizes how often each true class is predicted as each possible class. Detailed recognition results, including the confusion matrix, are reported together with other experimental outcomes in Section 6.

### 5.5. Baseline Models (For Comparison)

To contextualize the CNN performance, we additionally compare against conventional classifiers trained on the same input representation. Specifically, we consider standard baselines such as *k*-nearest neighbors (kNN) and support vector machines (SVM) using flattened pressure maps as features. These baseline results are reported in Section 6 to support discussion of model complexity versus performance.

## 6. Experiments and Results

This section evaluates the proposed operation support system using (1) recognition performance of the tactile object classifier, (2) task performance in terms of solving time and number of disc movements, (3) subjective workload using the NASA-TLX questionnaire, and (4) perceived usability using the System Usability Scale (SUS).

### 6.1. Participants and Experimental Design

Five adult participants (*n* = 5) from the laboratory took part in the study. Demographic variables (e.g., age and gender) were not recorded. Prior experience with the Tower of Hanoi task and mechanical interfaces was not formally assessed. All participants provided informed consent prior to participation. Participants were tested under normal working conditions; sleep, medication, and stimulant intake were not controlled and are listed as limitations.

A within-subject design was used with two conditions:

- **Baseline (no support):** Participants solved the Tower of Hanoi puzzle without assistance.
- **Support (proposed system):** Participants solved the puzzle using the tactile-glove-based guidance system.

Each participant completed both conditions, and the order was counterbalanced to reduce learning effects.

### 6.2. Task and Procedure

The task was a four-disc Tower of Hanoi puzzle (*n* = 4), for which the optimal solution requires 15 moves. In each condition, participants were instructed to move the full disc stack from the start peg to the target peg while following the rule that a larger disc cannot be placed on a smaller disc. In the support condition, the GUI presented step-by-step instructions and the system verified grasped disc identity and placement using combined glove and pressure-sheet signals.

Before the trials, participants received a brief explanation of the rules and completed a short familiarization period to reduce confusion unrelated to the interface. Each trial started when the participant initiated the first disc movement and ended when the stack was correctly completed on the target peg. After each condition, participants completed the NASA-TLX questionnaire (Appendix A). After finishing the support condition, participants completed the SUS questionnaire (Appendix B).

### 6.3. Recognition Performance

**Figure 24:**
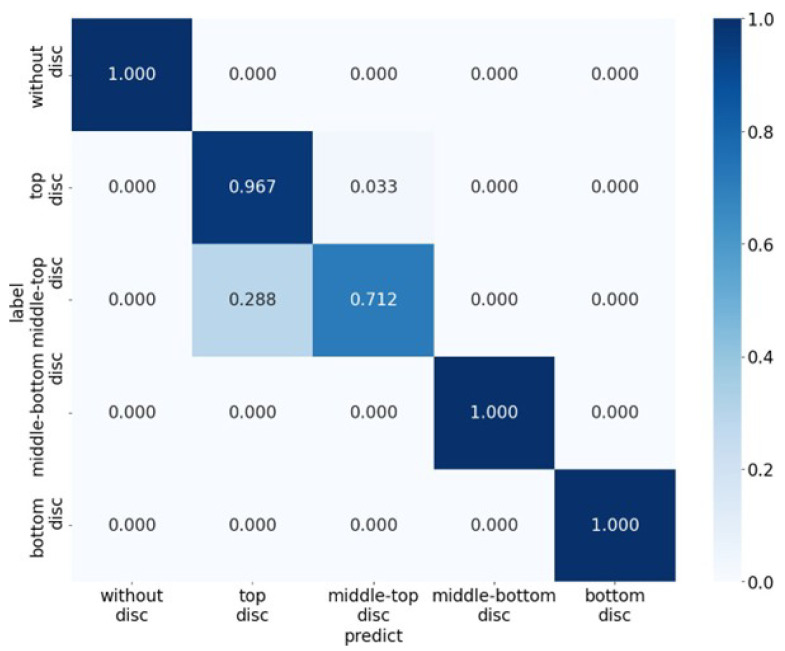
Confusion matrix of the CNN classifier on the test set. Rows correspond to true classes and columns to predicted classes.

The tactile classifier performance is summarized in Section 5. The final CNN achieved 93.3% accuracy on a separate-day test set recorded from a known user (see Section 4.1.1). Figure 24 shows the confusion matrix of the CNN classifier on the test set. Most errors occurred between the middle-top and middle-bottom discs, which have similar tactile contact patterns; however, recognition was sufficiently reliable for real-time verification within the guidance pipeline.

### 6.4. Task Performance: Solving Time

**Figure 25:**
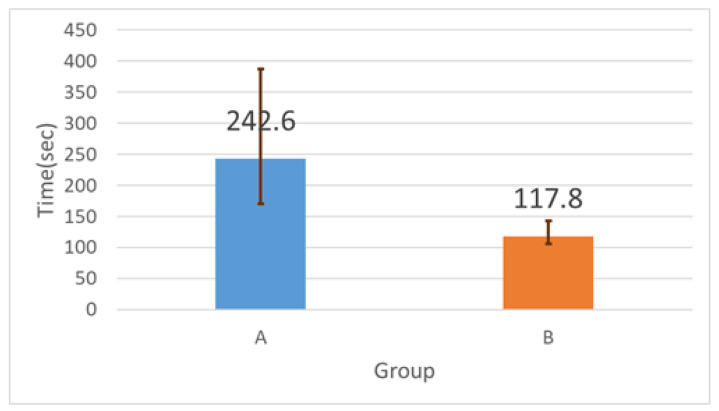
Total solving time for the baseline (no support) and support conditions. Error bars show standard deviation across participants.

Figure 25 shows the total solving time for the baseline and support conditions. The proposed system reduced mean solving time from 242.6 s (baseline) to 117.8 s (support), corresponding to a 51.5% reduction.

### 6.5. Task Performance: Number of Disc Movements

**Figure 26:**
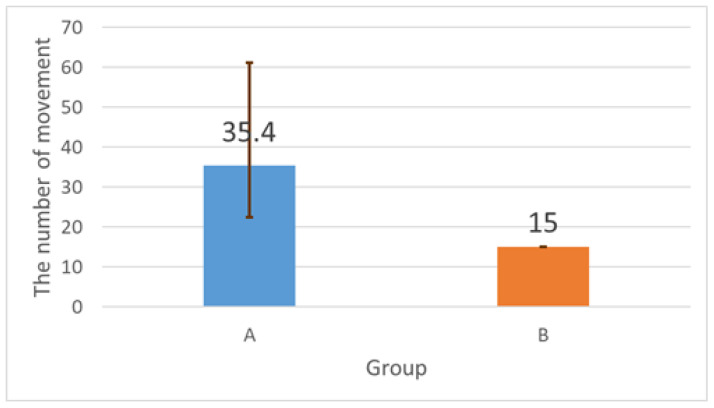
Number of disc movements in baseline vs. support conditions. The optimal number of moves is 15.

The optimal solution for a four-disc Tower of Hanoi puzzle requires 15 moves. Figure 26 shows that participants in the baseline condition frequently exceeded this number due to mistakes and inefficient reasoning. With system support, disc movements decreased from a mean of 35.4 (baseline) to 15 (support), i.e., 20.4 fewer moves on average (57.6% reduction), and were consistently close to the optimal solution.

### 6.6. Subjective Workload (NASA-TLX)

**Figure 27:**
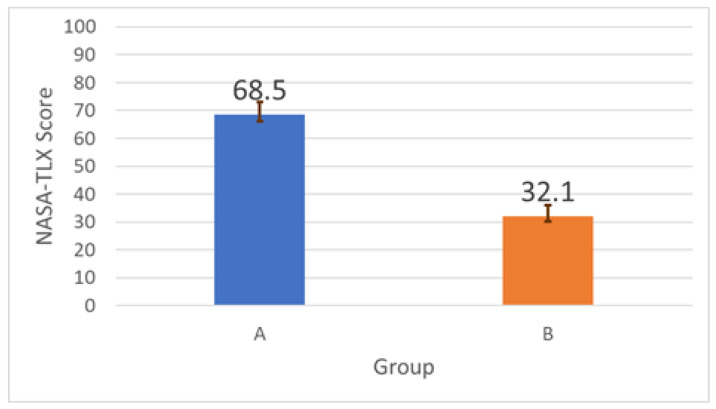
Overall NASA-TLX scores comparing baseline and support conditions. Lower is better.

Subjective workload was assessed using the NASA Task Load Index (NASA-TLX) [24], which includes six subscales: mental demand, physical demand, temporal demand, performance, effort, and frustration. Figure 27 shows that overall NASA-TLX decreased from 68.5 (baseline) to 32.1 (support), corresponding to a 53.1% reduction. Given the small sample size (*n* = 5) and uncontrolled participant preparation factors, these results are interpreted as indicative trends rather than definitive population-level estimates.

**Figure 28:**
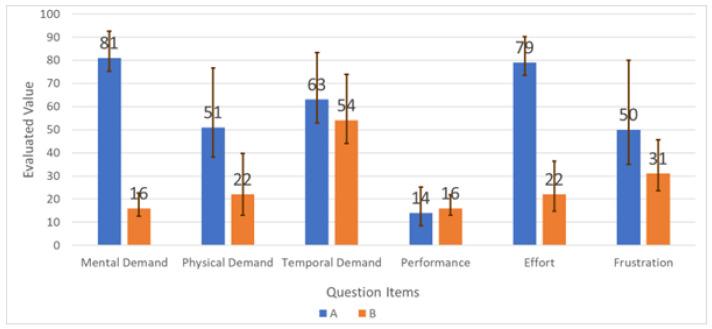
NASA-TLX subscale scores for baseline and support conditions.

**Figure 29:**
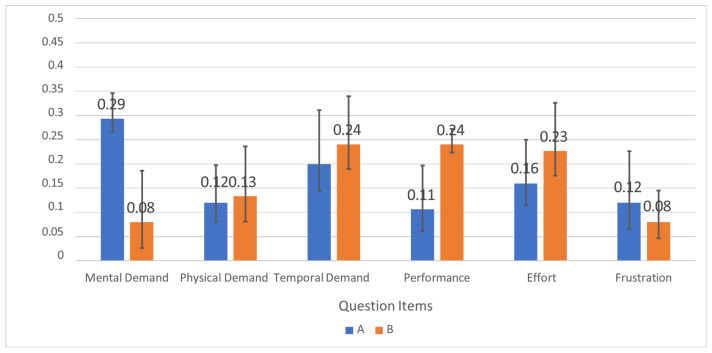
Weight distribution assigned by participants to each NASA-TLX category.

Figure 28 breaks down NASA-TLX subscale scores, and Figure 29 shows the user-assigned weights used to compute the final weighted score.

### 6.7. Usability Evaluation (SUS)

The System Usability Scale (SUS) [25] was administered for the support condition. The proposed system achieved an average score of

SUS=75

**Figure 30:**
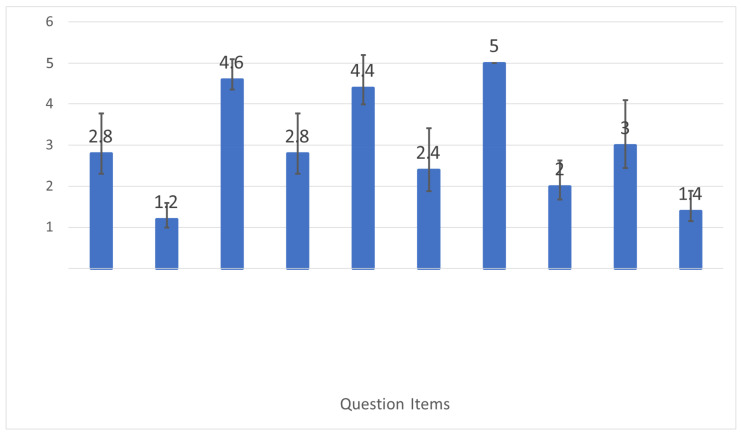
System Usability Scale (SUS) scores for the support system.

Figure 30 shows the SUS scores for the support system. A SUS score of 68 is commonly treated as a threshold for acceptable usability, suggesting that the system achieved good perceived usability in this study.

### 6.8. Summary of Experimental Findings

Across objective and subjective metrics, the proposed system improved performance in this controlled proxy task:

- 93.3% recognition accuracy on the separate-day test set.
- 51.5% reduction in mean solving time (242.6 s → 117.8 s).
- 57.6% reduction in disc movements (35.4 → 15), i.e., about 20 fewer moves on average.
- 53.1% reduction in NASA-TLX (68.5 → 32.1), interpreted as a trend given *n* = 5.
- SUS = 75, indicating good usability.

These findings support the feasibility of tactile-based step verification and guidance in a structured multi-step task, while broader generalization and real-world deployment require additional evaluation with larger and more diverse participant groups and tasks.

## 7. Discussion

The experimental results indicate that integrating tactile sensing with a real-time guidance interface can improve user performance in a structured multi-step manipulation task. This section discusses the implications of the findings, possible mechanisms behind the observed improvements, and the limitations that should be addressed in future work.

### 7.1. Effectiveness of Tactile Sensing for Task Guidance

The CNN classifier achieved an accuracy of 93.3%, which was sufficient for stable real-time operation in our guidance pipeline. Misclassifications occurred mainly between the middle-top and middle-bottom discs due to similar tactile profiles; however, such errors were infrequent and did not noticeably degrade usability in this controlled setting. The denoising procedure, which subtracts the average no-disc grasping pressure to emphasize the disc-specific contact patterns, likely contributed to the robustness of recognition.

A key advantage of tactile sensing is that it captures interaction patterns directly at the hand–object interface. This avoids common failure modes of vision-based systems such as occlusion, illumination variation, and camera-placement constraints. In the proposed system, direct contact sensing enabled closed-loop verification of grasp and placement events, supporting step-by-step procedural guidance.

### 7.2. Impact on Task Performance

Participants completed the puzzle substantially faster when using the support system. Mean solving time decreased from 242.6 s in the baseline condition to 117.8 s in the support condition (51.5% reduction). The number of disc movements also improved, decreasing from 35.4 to 15 moves on average (approximately 20 fewer moves), which is close to the optimal 15-move solution for a four-disc Tower of Hanoi.

These improvements suggest that the system reduced error-driven backtracking and hesitation by preventing invalid actions and providing immediate confirmation of correct progress. Importantly, the system did not replace human execution; rather, it acted as a procedural scaffold that reduced uncertainty and helped participants follow an efficient sequence.

### 7.3. Subjective Workload and User Perception

NASA-TLX results indicate a substantial reduction in perceived workload when using the support system (68.5 to 32.1; 53.1% reduction), where lower NASA-TLX scores generally reflect lower subjective workload [24]. Reductions were most apparent in mental demand and frustration, consistent with the system offloading rule checking and step validation. Because the study involved a small number of participants (*n* = 5), these results should be interpreted as indicative trends rather than definitive population-level estimates.

The SUS score of 75 reflects good overall usability. Participants reported that the GUI instructions were easy to follow and that real-time feedback was helpful for avoiding confusion and reducing trial-and-error behavior.

### 7.4. Potential Applications and Target Users

Although the Tower of Hanoi is a controlled proxy task, the underlying concept—closed-loop step verification and procedural guidance using contact sensing—is relevant to hands-busy, multi-step workflows where camera-based guidance can be unreliable due to occlusion, viewpoint constraints, or lighting [9,13,15,16]. Potential target users include trainees learning standardized manual procedures and operators performing stepwise tasks in settings such as assembly, inspection, or packaging, where correct sequence execution is critical and errors are costly [15,16]. In these scenarios, tactile sensing provides direct evidence of grasp and contact events, and the guidance interface can reduce uncertainty and improve procedural consistency by tracking hand–object interactions during task execution [9,13,21]. However, task-specific validation is required before making deployment claims for any particular industrial workflow.

### 7.5. Limitations

Several limitations should be acknowledged:

- **Limited participant pool and statistical power:** Only five individuals participated in the evaluation. While improvements were consistent in this sample, larger and more diverse participant groups are required to quantify variability and draw stronger statistical conclusions.
- **Learning and within-subject effects:** Each participant completed both conditions (counterbalanced order). Although counterbalancing reduces systematic learning bias, practice effects and familiarity with the puzzle may still influence performance.
- **Generalization of recognition:** The independent test set was collected from a participant who was also included in the training data, which primarily evaluates day-to-day repeatability for a known user rather than cross-user generalization. Future studies should include leave-one-subject-out evaluation and testing with unseen participants.
- **Similar disc classes:** The middle-top and middle-bottom discs produced overlapping tactile signatures, occasionally leading to misclassification. Additional sensing modalities (e.g., IMUs) or redesigned sensing layouts may reduce ambiguity.
- **Task domain:** The Tower of Hanoi provides a controlled proxy for multi-step manipulation with a deterministic optimal solution. It does not fully represent the variability and constraints of real operational tasks, so domain-specific validation is required before practical deployment claims can be made.
- **Hardware robustness:** The glove requires careful fabrication and calibration. Long-term durability, comfort over extended use, and robustness to sensor drift and wear were not evaluated in this study.

### 7.6. Future Work

Future work will focus on improving generalization and validating the approach in more realistic settings:

- **Broader evaluation:** Increase participant diversity and evaluate cross-user generalization using subject-independent splits (e.g., leave-one-subject-out).
- **Multi-modal fusion:** Combine tactile sensing with IMUs or other modalities to improve robustness for ambiguous grasps and complex interactions.
- **Task-level transfer:** Apply the framework to domain-specific multi-step procedures (e.g., assembly or inspection) to evaluate scalability and practical relevance.
- **Improved glove design:** Refine the tactile array layout and ergonomics, and evaluate long-term stability under repeated use.

Overall, the findings support the feasibility of tactile-based step verification and guidance for controlled multi-step tasks, while also highlighting the need for broader evaluation and domain-specific validation.

## 8. Conclusions

This study developed and evaluated a tactile data glove-based operation support system designed to guide users through the Tower of Hanoi puzzle as a controlled proxy for multi-step manipulation. The system integrates an e-textile glove with 88 pressure sensors, a tactile pressure sheet for placement verification, a CNN-based object classifier, and a graphical user interface that provides real-time, step-by-step instructions. By combining tactile sensing with procedural verification, the system recognizes user actions and helps prevent incorrect moves during the task.

Experimental results indicate that the proposed system improves task performance and user experience in this controlled setting. The CNN classifier achieved 93.3% recognition accuracy on a separate-day test set, enabling reliable identification of disc sizes and bare-hand states for real-time verification. In a within-subject user study with five participants, the system reduced mean solving time from 242.6 s to 117.8 s (51.5% reduction), reduced disc movements from 35.4 to 15 on average (about 20 fewer moves, close to the optimal 15-move solution), and lowered perceived workload (NASA-TLX) from 68.5 to 32.1 (53.1% reduction), while achieving a SUS score of 75, indicating good usability.

These findings support the feasibility of tactile-based step verification and guidance for structured multi-step tasks, particularly in scenarios where vision-based methods can be limited by occlusion or lighting conditions. However, broader generalization requires evaluation with larger and more diverse participant groups, subject-independent recognition tests, and validation on additional tasks beyond the Tower of Hanoi.

Future work will focus on improving cross-user generalization, enhancing glove robustness and ergonomics, and extending the guidance framework to domain-specific multi-step procedures.

## Figures and Tables

**Table 1:** Representative related work and comparison with the present study.

Study/Category	Sensing Modality	Task Type	Model/Method	Output Focus
Sundaram et al. [8]	Dense tactile glove (>500)	Everyday objects	CNN	Object recognition; estimation
Pohtongkam and Srinonchat [11]	Tactile glove	Objects	CNN vs. BoW	Accuracy vs. speed
Liu et al. [9]	IMUs + tactile	Manipulation	Visualization/analysis	Pose + pressure visualization
Smart glasses/XR [14,15,16]	Camera-based	Guidance/training	Vision + overlays	Visual guidance; detection
Luo et al. [22]	Conformal tactile textiles	Interaction sensing	Learning-based	Tactile interaction modeling
Murphy et al. [23]	Wireless tactile toolkit	Tactile sensing platform	Toolkit/framework	Data acquisition; prototyping
This work	Tactile glove (88) + pressure sheet	Multi-step manipulation (proxy)	CNN + verification logic	Step verification + procedural guidance

**Figure A1:**
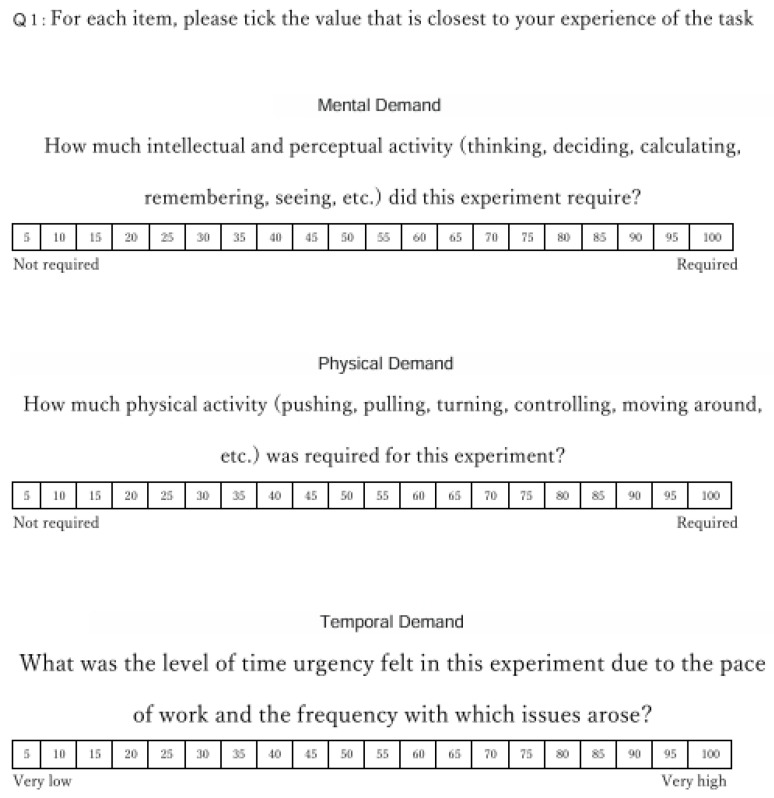
NASA-TLX questionnaire—Page 1.

**Figure A2:**
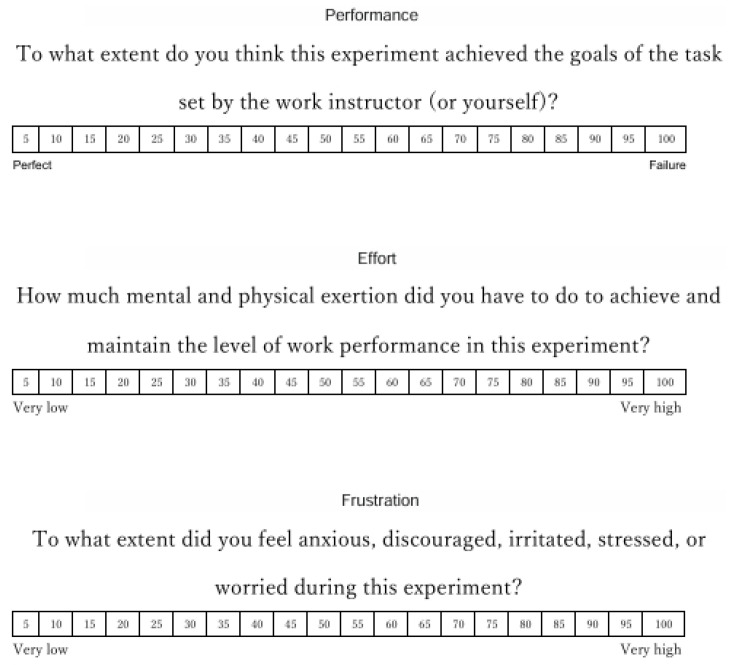
NASA-TLX questionnaire—Page 2.

**Figure A3:**
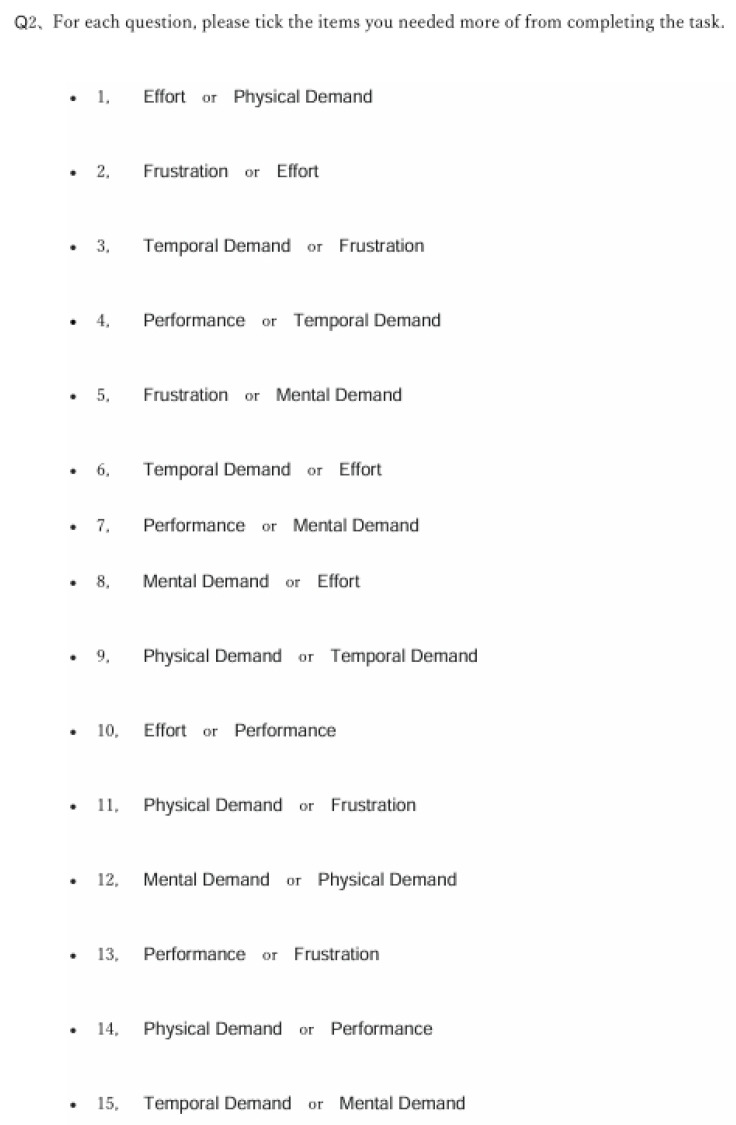
NASA-TLX questionnaire—Page 3.

**Figure A4:**
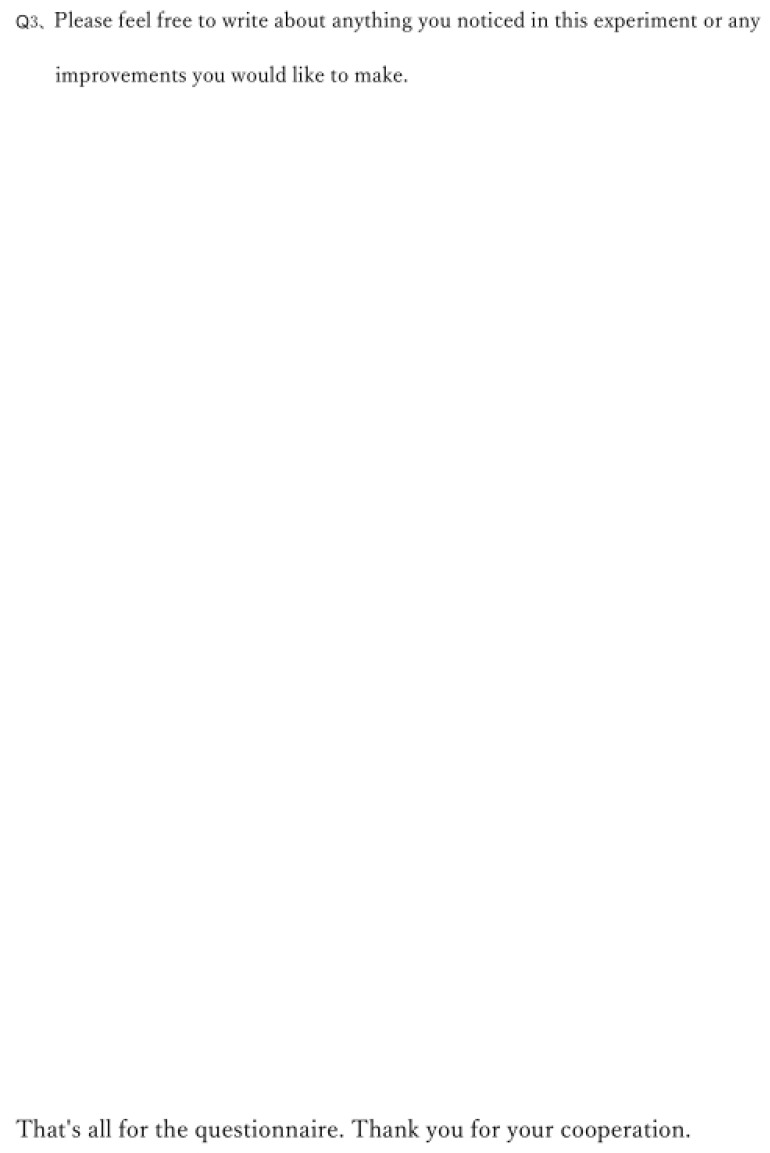
NASA-TLX questionnaire—Page 4.

**Figure A5:**
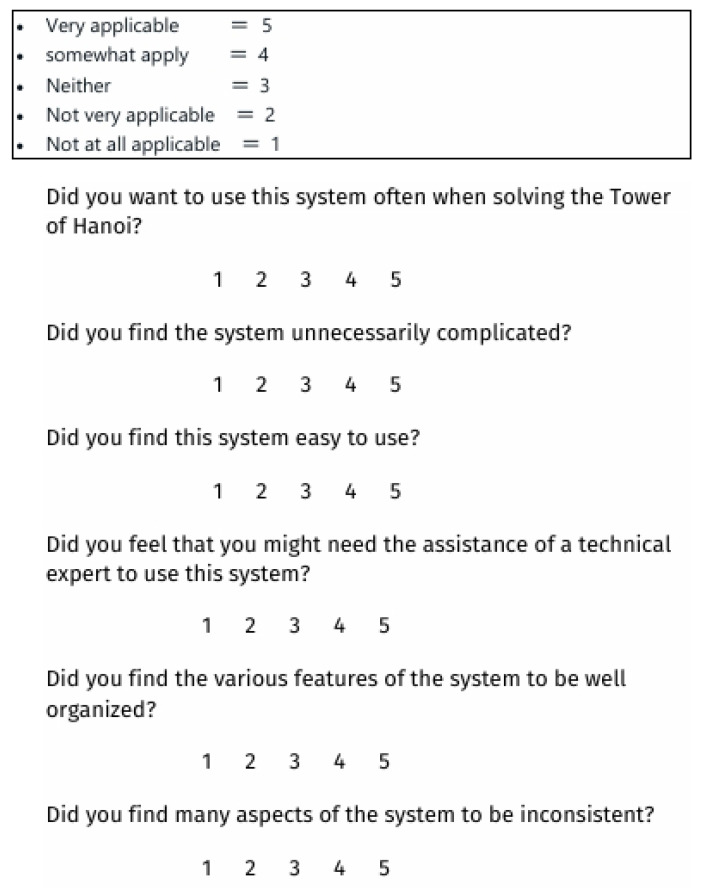
System Usability Scale (SUS) questionnaire—Page 1.

**Figure A6:**
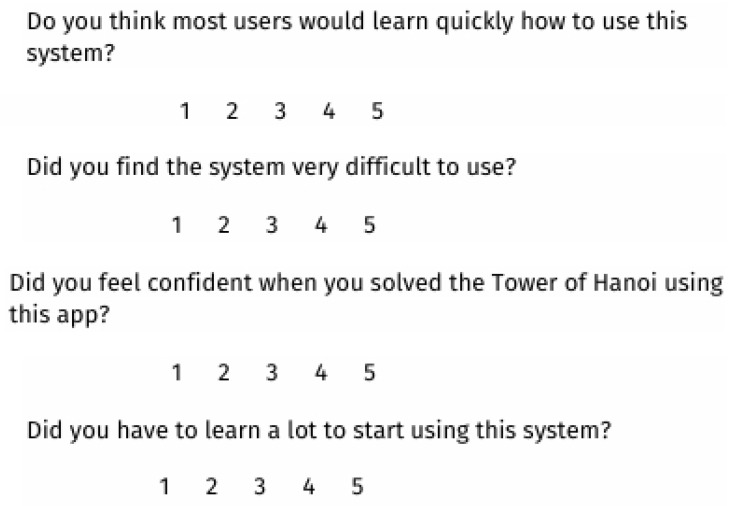
System Usability Scale (SUS) questionnaire—Page 2.

## Data Availability

The data presented in this study are available on request from the corresponding author. The data are not publicly available due to privacy considerations.

## Appendix A. NASA-TLX Questionnaire

The NASA Task Load Index (NASA-TLX) was used to assess subjective workload across six dimensions: mental demand, physical demand, temporal demand, performance, effort, and frustration. Participants completed the paper-based questionnaire after solving the Tower of Hanoi puzzle in both baseline and support conditions. The following pages show the exact NASA-TLX forms used in the study.

## Appendix B. System Usability Scale Questionnaire

The System Usability Scale (SUS) was administered for the support condition. The SUS consists of ten statements rated on a 5-point Likert scale and provides a global measure of perceived usability. The following pages show the SUS questionnaire used in the evaluation.
